# Sludge reduction by *lumbriculus variegatus* in Ahvas wastewater treatment plant

**DOI:** 10.1186/1735-2746-9-4

**Published:** 2012-08-02

**Authors:** Yalda Basim, Mahdi Farzadkia, Nematollah Jaafarzadeh, Tim Hendrickx

**Affiliations:** 1 Department of Environmental Engineering, Sazabpardazan Consultant Engineering, Ahvaz, Iran; 2 Department of Environmental Health Engineering, School of Public Health, Tehran University of Medical Sciences, Tehran, Iran; 3 Department of Environmental Health Engineering, School of Health, Ahvaz Jondishapour University of Medical Sciences, Ahvaz, Iran; 4 Department of Environmental Technology, Wageningen University, Wageningen, The Netherlands

**Keywords:** Sludge reduction, Aquatic worms, Ahvaz wastewater treatment plant, *Lumbriculus variegatus*

## Abstract

Sludge production is an avoidable problem arising from the treatment of wastewater. The sludge remained after municipal wastewater treatment contains considerable amounts of various contaminants and if is not properly handled and disposed, it may produce extensive health hazards. Application of aquatic worm is an approach to decrease the amount of biological waste sludge produced in wastewater treatment plants. In the present research reduction of the amount of waste sludge from Ahvaz wastewater treatment plant was studied with the aquatic worm *Lumbriculus variegatus* in a reactor concept. The sludge reduction in the reactor with worm was compared to sludge reduction in a blank reactor (without worm). The effects of changes in dissolved oxygen (DO) concentration up to 3 mg/L (run 1) and up to 6 mg/L (run 2) were studied in the worm and blank reactors. No meaningful relationship was found between DO concentration and the rate of total suspended solids reduction. The average sludge reductions were obtained as 32% (run 1) and 33% (run 2) in worm reactor and 16% (run 1) and 12% (run 2) in the blank reactor. These results showed that the worm reactors may reduce the waste sludge between 2 and 2.75 times higher than in the blank conditions. The obtained results showed that the worm reactor has a high potential for use in large-scale sludge processing.

## Introduction

Biological treatment is the most used technology in wastewater purification. Generation of large amounts of sludge is one the major features undertaken in the some biological wastewater treatment plants [1]. Such excess sludge has to be properly treated prior to final disposal, even though the cost of sludge treatment is extremely high, accounting for up to 60% of the total operating cost in a wastewater treatment plant [2].

At the present time, there are not any principle activities for pollution control of disposal sludge from municipal wastewater treatment plants (WWTPs) in Iran, and therefore it is generally unstable [3]. Reuse or discharging of raw sludge would lead to many hazardous materials which would pollute natural resources such as water, soil and agricultural products [4]. Activated sludge process is one of the most comprehensive and widely-used biological processes in domestic and industrial treatment plants in Iran [5]. This process is generated of organic and inorganic sludge to a great deal, that is considered a disadvantage [6]. Based on the results of the study that was done by Farzadkia in four local municipal wastewater treatment plants in Tehran which worked based on activated sludge process, except one of them, the others disposed untreated sludge [7].

Strict environmental regulations regarding waste sludge in wastewater treatment plants and the high expenditure of treatment, transportation and disposal of sludge have intensively led to the sludge minimization [5]. The important methods for the reduction of excess sludge are: endogenous metabolism, uncoupling metabolism, increase of dissolved oxygen in the reactor, oxic settling-anaerobic, ultrasonic cell disintegration, alkaline heat treatment, and growth of controllable predators. Also oxidation of a part of produced sludge is done by oxidizing materials such as chlorine and ozone [2,8].

Recently, the use of protozoa and metazoa has been proposed as a biological method for sludge reduction. This method, which is based on relationships present in the food chain and causes the overall reduction of the biomass, has gained more attention due to its low energy consumption and the lack of subsequent pollution [9]. A number of researches have been carried out on the reduction of disposed sludge by aquatic worms. Hendrickx *et al*. compared the performance of a species of aquatic worms called *Lumbriculus varigatus* in a reactor with perforated media containing worms with a usual reactor without any worms. The results of this research showed that the rate of total suspended solids (TSS) reduction in the reactor containing worms, in most of the cases, was three times greater than the reactor without worms [10].

In addition, Huang *et al.* studied the reduction of the sludge produced by activated sludge process by a species of aquatic worms called *Tubifex tubifex* in a reactor [11]. An ecological method regarding the use of four types of microfauna for the reduction of waste sludge has been surveyed by Liang *et al*. The results of this survey showed that the rate of sludge reduction depends on the classification and body size of the microfauna, and the species in the kingdom of *Oligochaeta*[9]. Wei *et al.* found out that worms, according to body size, are the biggest organisms in the sludge treatment cycle, compared with protozoa. They are easier to maintain and due to body size, have enough capability in sludge reduction [12]. Wei and Liu designed a combined reactor for the reduction of sludge using both free-flowing worms and sessile ones. In this experimental reactor, the TSS of sludge was reduced by 48 percent, which is mainly due to the presence of *Tubificidea*[13]. Rastak studied the possibility of reducing activated sludge in wastewater treatment plants using aquatic worms of *Oligochaeta* at experimental scales, and the environmental factors affecting the performance of worms were surveyed. This research confirmed the use of worms as a protein-supply source in the food for fish and domestic animals [14].

Although the advantages of aquatic worm application for sludge reduction are known, this method has not yet been studied in any scale in Iran. The objectives of this study were identification of appropriate and endemic species of aquatic worm called *Lambriculus variegates*, adaptation of life natural environment of worms to laboratory conditions, determination of sludge reduction by *Lambriculus variegates* worm in Ahvaz Wastewater Treatment Plant (WWTP) and determination of effective DO concentration in worm reactor. Ahvaz WWTP operated based on activated sludge process for wastewater treatment and has two stages of anaerobic digester for sludge stabilization. The first stage of anaerobic digester was damaged and was out of service at the time of this study. Comparison of the microbial quality of disposal sludge in Ahvaz WWTP and USEPA criteria showed that the sludge was not in class A or B conditions. Hence, it should not be disposed to the environment or reused for any purpose [15].

## Materials and Methods

Provision of the worms from the specified species was a chief concern in this research. Due to the possibility of access to this species in areas with organic and decomposed materials, the probable homes of this species were identified. Samples were taken from the benthic materials of Khuzestan water reservoirs including Karoon River, Maleh Stream, Shadegan Wetland, and Dez river by using grab model of Van Vee [10]. Microscopic pictures of the samples were then taken and identified with “Freshwater Biology” [16]. No worms belonging to the subclass *Lumbiculidae* were observed.

Due to the study carried out on the spring pools of Kermanshah province in the west part of Iran [17], a few samples were taken and observed in this area. Finally, the species of *Lumbriculus variegates* from the *Oligochaeta* subclass and *Lumbiculidae* family were found in the sediments deposited of Jabery and Ravansar springs. To maintain the environment of the worms collected as well as their compatibility with the new environment, the vessels containing worms were aerated, and the temperature of the medium was constantly controlled [18]. In order to take samples of aquatic worm species and to carry out field tours, a collection of outdoor equipment including the Sampler VN, a GPS, electrical conductivity meter, mercury and digital thermometers, kit of DO analyzer, a sieve with one millimeter mesh, and other equipments for transportation of samples were used [14].

The worm reactor consisted of a beaker containing both waste sludge and worms. It contained 1500 mg wet weight of the aquatic worm and 100 mL of waste sludge of Ahvaz WWTP. The blank reactor was investigated under the same conditions but without any worm. The waste sludge was daily provided from the return line of Choneibeh WWTP in the west of Ahvaz city. Experiments were carried out in two parallel runs with two different dissolved oxygen contents. Each run consisted of 8 batch steps and took 1 day. Therefore, each run took 8 days; repetition of each batch step was done 8 times. The concentration of the dissolved oxygen in steps one and two were kept up to 3 and 6 mg/L, respectively [10]. pH, temperature and TSS of the incoming and outgoing sludge were determined every 24 hours at the end of each step.

Every 24 hours, aeration was stopped for a short time and, by using a fine mesh; the worms were separated and prepared for entering to the next batch step [14]. The species of the aquatic worms during the days of the experiment were similar: they were selected among the same initial population under the experiment [10]. All experiments were carried out by using the standard methods [19], and the results were assessed based on ANOVA analysis and T statistical test in order to investigate the average differences at a reliable level of 95%. The Smirnov-Kolmogrove test was used to determine the normal distribution of the findings. The effect of water evaporation in aeration was noticed via adding sample volume about 20% in the start time. Selection of 20% was based on the pretests.

## Results

**Figure 1:**
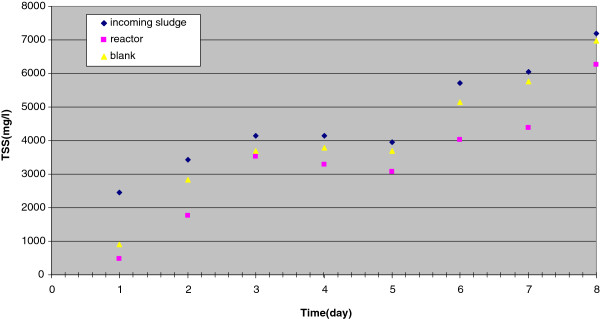
TSS concentration in reactor and blank-Run 1.

**Figure 2:**
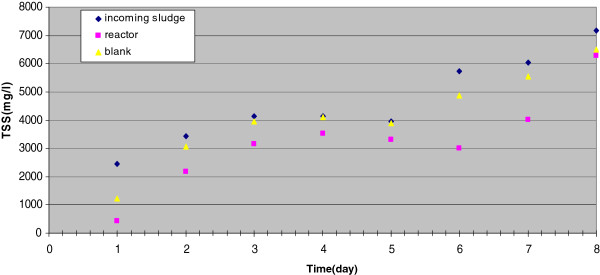
TSS concentration in reactor and blank-Run 2.

**Figure 3:**
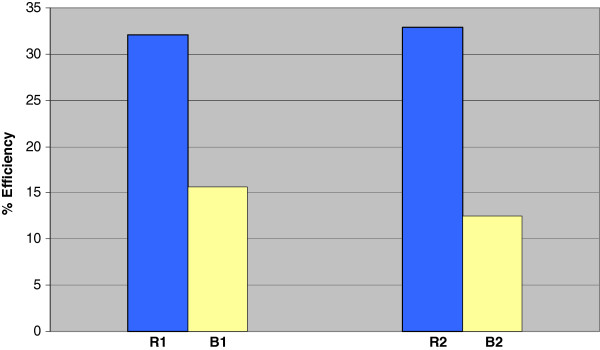
Efficiency of sludge reduction in the reactors (R) and blanks (B) in runs (1) and (2).

TSS in the incoming and outgoing sludge of the reactors and blanks are indicated in Figure 1 and Figure 2, which show that, during the whole days of the two runs, TSS in outgoing sludge was less than that the incoming sludge. The minimum and maximum amounts of TSS of sludge entry the reactors and blanks were 2456 and 7184 mg/L, respectively. The efficiency of sludge reduction in the reactors, as well as the blanks are shown on Figure 3, which indicates that the efficiency of sludge reduction in the second-phase reactor was 33%, slightly higher than 32% of the first-phase reactor. The rates of sludge reduction in the blank reactors in the two phases were 16% and 12%, respectively. The average sludge reduction by the species *Lumbriculus variegatus* was found to be about 0.45 mg of sludge per one mg of the worm per day. The Smirinove- Kolmogrove test on the suspended solids in the sludge and the dissolved oxygen showed that the data followed a normal distribution. Based on the data obtained from runs (1) and (2) in the reactors, no significant difference was observed between the sludge reductions in two runs (p<0.05). These results were confirmed by the sludge reduction in two runs in the blanks (p<0.05). In other words, variation of oxygen concentration did not affect the sludge reduction efficiency in these experiments.

However, statistical analyses indicated that there is a meaningful difference between the rates of sludge reduction in the reactors with the blanks in two runs (p>0.05). Due to similar medium conditions of the reactors and the blanks, this meaningful difference highly confirmed the effectiveness of the worms in the reactors for sludge reduction.

## Discussion

In the present study, the rate of sludge reduction in the first and second runs of the reactors were 32% and 33% and in the runs (1) and (2) of blanks were 16% and 12%, respectively. In the reactor containing the aquatic worms of *Lumbriculus variegatus*, which were experimented by Hendrickx *et al*., the rate of sludge reduction was almost three times higher than in the blank (without worms) [10]. In the present study, the sludge reduction rate in the worm reactors were 2 to 2.75 times higher than in the blank. This difference may be based on the differences between the methods of experiments in two researches. In another survey in China, sludge reduction was studied by using of a combination of protozoa and metazoa and the rate of reduction (in MLSS) was from 45% to 58%, compared to that of the blank. In this survey, the DO concentration and hydraulic retention time of sludge were considered as 1–4 mg/L and 6–13 hours, respectively [20]. The higher efficiency of sludge reduction in this study may be resulted from the use of a combination of protozoa and metazoa in one reactor.

In another study, carried out in China on the aquatic worm species of *Tubificide*, the average TSS reduction in the sludge was 48% and the DO concentration was ≥2 mg/L [13]. A comparison of the 48% reduction of the above-mentioned research with that of the present research (33%) shows that the aquatic worm *Tubificide* has a better performance than the species of *Lumbriculus variegatus*. It may be noted that, Wei and Liu alternately used two reactors as series containing worms.

In another research which was performed in the Netherlands in an aerobic reactor on the activated sludge containing worm species of *Lumbriculus variegates*[14], almost similar result for sludge reduction (up to 30%) compared to our study (33%), was obtained, which is because of similar worm species and treatment method (activated sludge). In China, another survey was carried out by Guo *et al.,*[21] in which, a reactor containing worm species of *Tubificide* was used to reduce sludge and an efficiency of 46.4% was obtained. The SRT (Solid Retention Time), HRT (Hydraulic Retention Time) and DO concentration in this research were 30 days, 15.4 hours, and 0.5-3 mg/L, respectively [21]. Better results in this study may be related to longer SRT (4 times more) and type of the worms (*Tubificide*).

Wei *et al*, showed that the performance of the worm species *Oligochaeta* in the conventional activated sludge (CAS) system could lead to considerable reduction in sludge and improve its settling characteristics [22]. The similar results of sludge reduction was obtained in our study on Ahvaz WWTP with CAS process.

The daily rate of sludge reduction by four species of worms was studied by Liang *et al*[9]. These rates were 0.1 to 0.54 mg sludge/mg worm, based on the order or body size of the worm [9]. The average sludge reduction in our study (0.45 mg sludge/mg worm per day) is almost near to the maximum rate of sludge reduction in the above-mentioned research.

The quantitative results of this research, regarding the sludge reduction by worm species *Lumbriculus variegatus*, were in agreement with Hendrickx's study (36%) [23]. But, no meaningful relationship was found in our research between DO concentration and the rate of sludge reduction. This disagreement may be arising from differences in worm habitats and their living conditions, the type of reactors and operational conditions.

## Competing interests

The authors declare that they have no competing interests.

## Authors’ contributions

**YB:** conception and design, generation of data, collection of data, assembly of data, analysis of data, interpretation of data, drafting of the manuscript. **MF:** conception and design, interpretation of data, drafting of the manuscript, revision of the manuscript, approval of the manuscript. **NJ:** interpretation of data, drafting of the manuscript, revision of the manuscript, approval of the manuscript. **TH:** conception and design, revision of the manuscript, approval of the manuscript. All authors read and approved the final manuscript.
